# Underestimated anxiety in chronic diseases: A cross-sectional study on specific risk factors

**DOI:** 10.1097/MD.0000000000041791

**Published:** 2025-03-07

**Authors:** Yun Chen, Chaoer Wu, Wei Qian

**Affiliations:** a Department of General Practice, The First People’s Hospital of Hangzhou Lin’an District, Hangzhou Medical College, Hangzhou, Zhejiang Province, China.

**Keywords:** anxiety, chronic disease, mental disorder, risk factor, sociodemographic factor

## Abstract

Globally, approximately one-third of adults are affected by 1 or multiple chronic diseases, posing a considerable threat to mental health, particularly in relation to mental disorders. The objective of our study was to investigate the associations between chronic diseases and anxiety levels, as well as to identify the risk factors that contribute to the occurrence of anxiety in patients with chronic diseases. This cross-sectional survey was conducted from January 1, 2023, to January 1, 2024 at the First People’s Hospital of Hangzhou Lin’an District. The anxiety status was assessed by the tool of Zung Self-Rating Anxiety (SAS). A cohort of 50 patients was identified, among whom 23 (46%) were assessed as having anxiety, while 27 (54%) did not exhibit anxiety. Patients with anxiety had a higher level of existed in living alone compared to those without anxiety (*P* = .032). Notably, the SAS score of anxiety for patients with 4 chronic diseases (60.0 ± 11.2) was significantly higher compared to those with 1 chronic disease (41.2 ± 15.4, *P* = .034) and 2 chronic diseases (44.2 ± 13.2, *P* = .037). The analysis revealed that patients living alone were more likely to experience symptoms of anxiety compared to those living with family (odds ratio = 5.469; 95% confidence interval, 1.007–29.701, *P* = .049). Our findings substantiate the association between anxiety and chronic diseases. The significance of demographic factors in the manifestation of anxiety related to chronic diseases warrants further investigation.

## 1. Introduction

Globally, approximately 1 in 3 adults live with 1 or multiple chronic diseases, posing a significant health threat and increasing social and economic burdens.^[1]^ A number of chronic diseases affect a substantial portion of the population, including hypertension, diabetes mellitus, cardiovascular diseases, chronic obstructive pulmonary disease, stroke, and hyperlipidemia.^[2]^ Furthermore, patients with chronic diseases are at a greater risk than the general population for developing mental disorders, particularly anxiety and depression.^[3]^

The National Comorbidity Survey Replication conducted in America identified that a minimum of 68% of individuals diagnosed with a psychiatric disorder also presented with at least 1 comorbid general medical condition.^[4]^ Concurrently, an international study encompassing 41,810 adults demonstrated a significant correlation between mental disorders and chronic diseases, including diabetes, arthritis, asthma, chronic lung disease, angina, and stroke.^[5]^ In addition, the presence of mental disorders in patients with chronic diseases can lead to adverse outcomes such as diminished adherence to treatment regimens, increased healthcare costs, and elevated mortality rates.^[6,7]^ Thus, there is considerable interest in methods to facilitate psychological adjustment following the onset of chronic diseases.

Luckily, evidence has indicated that the development of chronic diseases is associated with a growing set of risk factors, including female, advanced age, poor sleep quality, lower educational attainment, and lower income.^[8–10]^ On the one hand, by understanding these existing risk factors, physicians may be better equipped to identify patients with chronic diseases who are likely to develop mental disorders. On the other hand, it facilitates early clinical interventions that have the potential to diminish the occurrence and development of anxiety in patients with chronic diseases.

The objective of our study was to investigate the associations between chronic diseases and anxiety levels, as well as to identify the risk factors contributing to the occurrence of anxiety in patients with chronic diseases.

## 2. Materials and methods

### 2.1. Study design and participants

This cross-sectional survey was conducted from January 1, 2023, to January 1, 2024 at the First People’s Hospital of Hangzhou Lin’an District. Participants were eligible for enrollment if they satisfied the following criteria: provide informed consent to participate in the study; were over 18 years old; underwent general medical examinations, including history inquiry, physical examination, questionnaire survey and laboratory tests; had at least 1 chronic disease. We also excluded participants based on the following criteria: refusal to participate in the study; recent cardiovascular events within the past 3 months, cerebrovascular events, uncontrolled severe hypertension, poorly controlled diabetes, other established mental or neurological disorders, and surgical operations performed within the last month. Ultimately, a total of 60 participants who met the inclusion were enrolled in the study. All participants provided informed consent by signing the consent forms. This study received approval from the Ethics Committee of the First People’s Hospital of Hangzhou Lin’an District.

### 2.2. Measurement

#### 2.2.1. Definition of chronic disease

Chronic disease was operationally defined to include any of the following conditions: hypertension, diabetes, cardiovascular disease, cerebrovascular disease, chronic lung disease, rheumatic disease, chronic digestive disease, chronic kidney disease or malignant tumor. To qualify for inclusion, all the patients were required to have a minimum diagnostic duration of 6 months. The diagnosis of chronic disease was corroborated through a joint assessment conducted by 2 senior physicians.

#### 2.2.2. Data collection

Personal demographic characteristics were collected, encompassing age, gender (male/female), marital status (married, unmarried), occupation (employed, unemployed, retired), place of residence (village, town), living status (alone, with family), and educational (primary or below, secondary school, college or above).

#### 2.2.3. Zung Self-Rating Anxiety (SAS)

The Self-Rating Anxiety (SAS) was employed to evaluate the presence and severity of anxiety among participants. The analysis was conducted by a psychiatrist with 15 years of clinical experience.^[11]^ The Zung SAS is a self-report instrument comprising 20 items that encompass a range of anxiety symptoms, including psychological manifestations (e.g., “I feel afraid for no reason at all” and “I feel like I’m falling apart and going to pieces”) and somatic manifestations (e.g., “My arms and legs shake and tremble” and “I feel my heart beating fast”). Responses are recorded on a 4-point scale Likert scale, ranging from 1 (none, or a little of the time) to 4 (most, or all of the time). Participants are instructed to base their responses on their experiences over the past week. The items include both negative and positive experience (e.g., “I fall asleep easily and get a good night’s sleep”), with the latter being reverse scored. The raw scale scores for the SAS span from 20 to 80. The SAS demonstrates satisfactory psychometric properties.

### 2.3. Statistical analyses

Data analysis was conducted utilizing SPSS statistical software version 26.0. Categorical variables were compared between groups using χ^2^ tests or Fisher’s exact tests. For continuous 2-sample independent data, either *t* test or ANOVA analysis was employed. The data met the normality criterion. Continuous data are represented as mean ± standard deviation. A multivariate logistic regression analysis was conducted to identify variables related to anxiety independently associated with anxiety. The results are reported as odds ratios (OR) with 95% confidence intervals (CIs) and *P* values. All statistical tests significant when the *P* value was <.05. All statistical tests were 2-tailed.

## 3. Results

### 3.1. Patient characteristics

**Figure 1.**
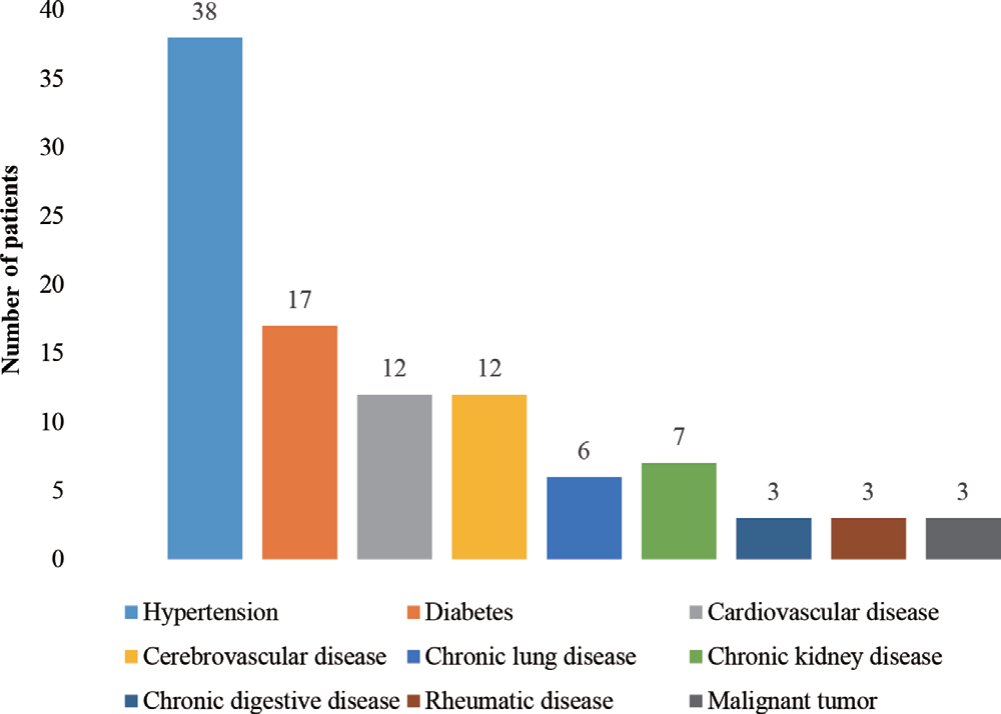
The spectrum of chronic diseases.

As illustrated in Table 1, a cohort of 50 patients was identified, among whom 23 (46%) were assessed with anxiety, while 27 (54%) did not exhibit anxiety. As depicted in Table 1, indicates that 20 (40%) of the patients were male and 30 (60%) were female, with a mean average age of 67.2 ± 12.9 years old. Notably, patients with anxiety had a higher level of existed in living alone compared to those without anxiety (*P* = .032). No statistically significant differences were observed in other demographic variables (*P* > .05). The mean number of comorbidities was 2.0 (interquartile range: 1.0, 3.0), with an average duration of 9.6 ± 7.1 years. The anxiety scores were assessed using the SAS, revealing significantly higher scores in the anxiety group compared to the non-anxiety group (*P* < .001). As shown in Figure 1, among the 50 participants with chronic diseases, the prevalence of specific conditions was as follows: hypertension (38 cases), diabetes (17 cases), cardiovascular disease (12 cases), cerebrovascular disease (12 cases), chronic lung disease (6 cases), chronic kidney disease (7 cases), chronic digestive disease (3 cases), rheumatic disease (3 cases), malignant tumor (3 cases).

**Table 1:** Baseline comparisons of the study arms.

Variables	All patients (n = 50)	Patients with anxiety (n = 23)	Patients without anxiety (n = 27)	*P* value
Demographic				
Age (yr)	67.2 ± 12.9	67.0 ± 9.7	67.5 ± 16.2	.910
Gender				
Male (n, %)	20 (40.0)	9 (39.1)	11 (40.7)	.908
Female (n, %)	30 (60.0)	14 (60.9)	16 (59.3)	
Marital status				
Married (n, %)	47 (94.0)	22 (95.7)	25 (92.6)	.650
Unmarried (n, %)	3 (6.0)	1 (4.3)	2 (7.4)	
Occupation				
Employed (n, %)	22 (44.0)	9 (39.1)	13 (48.1)	.721
Unemployed (n, %)	5 (10.0)	3 (13.0)	2 (7.4)	
Retired (n, %)	23 (46.0)	11 (47.8)	12 (44.4)	
Place of residence				
Village (n, %)	33 (66.0)	15 (65.2)	18 (66.7)	.914
Town (n, %)	17 (34.0)	8 (34.8)	9 (33.3)	
Living status				
Alone (n, %)	9 (18.0)	7 (30.4)	2 (7.4)	**.032**
With family (n, %)	41 (82.0)	16 (69.6)	25 (92.6)	
Education				
Primary or below (n, %)	29 (58.0)	14 (60.9)	15 (55.6)	.189
Secondary school (n, %)	19 (38.0)	8 (34.8)	11 (40.7)	
College or above (n, %)	2 (4.0)	1 (4.3)	1 (3.7)	
Chronic diseases				
Duration time (yr)	9.6 ± 7.1	10.4 ± 7.0	8.9 ± 7.2	.484
Number of chronic diseases (n, %)	2.0 (1.0, 3.0)	2.3 (1.0, 3.0)	1.5 (1.0, 2.0)	.288
Hypertension (n, %)	38 (76.0)	18 (78.3)	20 (74.1)	.730
Diabetes (n, %)	17 (34.0)	7 (30.4)	10 (37.0)	.623
Cardiovascular disease (n, %)	12 (24.0)	6 (26.1)	6 (22.2)	.750
Cerebrovascular disease (n, %)	12 (24.0)	6 (26.1)	6 (22.2)	.750
Chronic lung disease (n, %)	6 (12.0)	3 (13.0)	3 (11.1)	.834
Chronic kidney disease (n, %)	7 (14.0)	5 (21.7)	2 (7.4)	.225
Chronic digestive disease (n, %)	3 (6.0)	1 (4.3)	2 (7.4)	1.000
Rheumatic disease (n, %)	3 (6.0)	1 (4.3)	2 (7.4)	1.000
Malignant tumor (n, %)	3 (6.0)	3 (13.0)	0 (0)	.090
Anxiety assessment				
Anxiety score (SAS)	45.7 ± 14.2	58.5 ± 6.7	34.8 ± 8.6	**<.001**

Bold values indicate *P* < .05.

SAS = Self-rating Anxiety Scale.

### 3.2. Correlation analysis between chronic diseases and anxiety level

**Figure 2.**
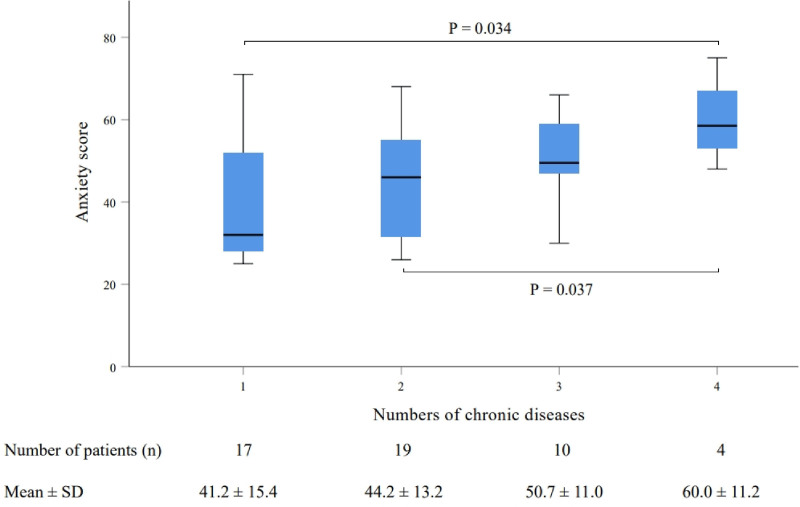
Anxiety score in patients with different numbers of chronic diseases. SD = standard deviation.

As illustrated in Figure 2, the distribution of patients with 1, 2, 3, and 4 chronic diseases was 17 (34%), 19 (38%), 10 (20%), and 4 (8%), respectively. An increase in the number of chronic diseases corresponded with a rise in the mean anxiety scores. Notably, the SAS score of anxiety for patients with 4 chronic diseases (60.0 ± 11.2) was significantly higher compared to those with 1 chronic disease (41.2 ± 15.4, *P* = .034) and 2 chronic diseases (44.2 ± 13.2, *P* = .037).

### 3.3. Factors associated with anxiety in participants

To investigate the influence of various factors such as age, gender, marital status, occupation, place of residence, living status, education, duration time, and the number of chronic diseases, a regression analysis was conducted. The results of the multivariate logistic regression analyses examining anxiety among the participants are presented in Table 2. The analysis revealed that patients living alone were more likely to experience symptoms of anxiety compared to those living with family (OR = 5.469; 95% CI, 1.007–29.701, *P* = .049). However, no significant associations were found for the other variables with in our cohort.

**Table 2:** Multivariate logistic regression analysis of factors associated with anxiety in chronic diseases.

Variables	Multivariate logistic regression (n = 50)	
	OR (95% CI)	*P*
Age	1.003 (0.960, 1.047)	.896
Gender		
Male	0.935 (0.300, 2.912)	.908
Female	Ref	
Marriage		
Married	0.404 (0.034, 4.767)	.472
Unmarried	Ref	
Occupation		
Employed	0.462 (0.04, 3.345)	.462
Unemployed	0.611 (0.085, 4.371)	.624
Retired	Ref	
Place of residence		
Village	1.067 (0.330, 3.448)	.914
Town	Ref	
Living status		
Alone	**5.469 (1.007, 29.701**)	**.049**
With family	Ref	
Education		
Primary or below	0.923 (0.053, 16.394)	.962
Secondary school	0.727 (0.039, 13.452)	.831
College or above	Ref	
Duration time	1.029 (0.950, 1.115)	.477
Number of chronic diseases	1.399 (0.758, 2.583)	.283

Bold values indicate *P* < .05.

CI = confidence interval, OR = odd ratio.

## 4. Discussion

In this study, we analyzed the relationship between chronic disease and anxiety levels in adult patients. Our findings indicate that the incidence of anxiety among patients with chronic diseases is 46%. Furthermore, there is a positive correlation between the number of comorbidities and the severity of anxiety symptoms. Logistic regression analysis revealed that patients who live alone are more likely to experience heighted anxiety symptoms compared to those who reside with family members.

Over recent decades, advancements in medical care and quality in China have led to an aging population and a concomitant rise in the prevalence of common diseases among adults.^[12]^ A cross-sectional survey of 2584 patients revealed a 14% prevalence of generalized anxiety disorder, with a higher incidence observed in females compared to males.^[13]^ Furthermore, a comparable study indicated that 15% to 23% of cancer patients exhibited anxiety symptoms; with the prevalence escalating to as 69% as the disease progressed.^[8]^ Meanwhile, a systematic review of 31 studies encompassing 16,922 participants demonstrated that individuals with anxiety or depression exhibit significantly higher physical symptoms and greater burdens of chronic disease compared to those without anxiety or depression.^[14]^ In our cohort, we observed a positive correlation between the number of chronic diseases and the mean anxiety score, indicating that as the number of chronic conditions increased, the mean anxiety score also rose.

The SAS, developed by Professor Zung in 1971, is extensively utilized for assessing anxiety symptoms in adults both domestically and internationally. However, the SAS has notable limitations. Firstly, the overlap of certain somatic symptoms (e.g., respiratory, cardiovascular, and urinary symptoms) in individuals with chronic illnesses can lead to an overestimation of anxiety prevalence in this population. Secondly, the SAS primarily depends on patient self-reports, which lack objectivity and cannot replace comprehensive psychiatric evaluations conducted by clinicians.^[11]^ However, as a longstanding and user-friendly clinical instrument, SAS software is integral to the initial screening process for anxiety, particularly aiding patients with chronic illnesses in early detection and facilitating enhanced assessments by professional psychiatrists at subsequent stages. Conversely, the hospital anxiety and depression scale (HADS) scale, another frequently employed tool for anxiety assessment in clinical settings, often underestimates anxiety prevalence due to its exclusion of physical symptoms and emphasis on cognitive dimensions.^[15]^ Consequently, the SAS scale is preferred for screening purposes in specific populations in our study.

The primary objective is to investigate the potential association between chronic diseases and anxiety. Anxiety, characterized by a feeling of uncertainty, fear, resentment, and frustration, can be conceptualized as a form of trauma devoid of both external and internal value.^[16]^ This condition may be exacerbated by the prolonged management of chronic diseases. The chronic and enduring nature of these illnesses often results in patients experiencing increased anxiety due to their progressively diminished ability to manage their health effectively. Furthermore, financial constraints pose significant challenges for many patients, resulting in poor medication adherence and adverse health outcomes.^[17]^ However, when clinicians identify risk factors associated with anxiety in patients with chronic diseases, they can implement early interventions to mitigate the occurrence of anxiety, thereby reducing the likelihood of developing subsequent mental disorders. Fortunately, evidence suggests that the development of chronic diseases is linked to an increasing array of risk factors, particularly social determinants. Previous studies have identified several risk factors associated with mental disorders, including living alone, social isolation, lack of social support, socioeconomic disadvantage, lower educational attainment, and other sociodemographic factors.^[9,10,18–20]^ In our model, we identified living alone as a risk factor positively correlated with the prevalence of anxiety among patients with chronic diseases. Unlike clinical factors, social demographics, which persist throughout a patient’s lifetime, present significant challenges for physicians to manage effectively.

In recent decades, numerous clinical trials have demonstrated the efficacy of psychotherapy in treating a wide range of mental illnesses.^[21]^ Supportive psychotherapy for patients with chronic diseases is predicated on addressing the unstable psychological adaptation processes that emerge following the onset of chronic illness. A common intervention technique involves managing the patient’s secondary frustrations, particularly their thoughts of “why am I so sick and not someone else.” Additionally, supportive psychotherapy is effective in addressing both acute and long-term conflict situations experienced by these patients.^[22]^ Therapists function as active listeners, typically maintaining silence while providing encouragement, thereby empowering patients to take initiative. They intervene solely to assist patients in recognizing and tolerating their emotions. This approach fosters an environment where patients are encouraged to be curious and compassionate, and to strive for a mutual understanding of their emotional state.^[23]^ Generally, psychotherapy does not impose a substantial financial burden on individuals with mental disorders, yet its clinical outcomes are notably significant.

## 5. Limitations

This study is subject to several limitations. Firstly, the cross-sectional design employed precludes the ability to ascertain causal relationships over time. Secondly, the exclusive use of a single scale may introduce measurement bias. Lastly, the effects of psychotherapy on the participants were not assessed.

## 6. Conclusions

Our findings substantiate the association between anxiety and chronic diseases. The significance of demographic factors in the manifestation of anxiety related to chronic diseases warrants further investigation. We advocate for additional research to explore innovative treatments for mental disorders in patients with chronic diseases.

## Acknowledgments

The authors thank all the research staﬀ for their team collaboration work.

## Author contributions

**Conceptualization:** Yun Chen, Chaoer Wu, Wei Qian.

**Data curation:** Yun Chen, Chaoer Wu, Wei Qian.

**Formal analysis:** Yun Chen, Chaoer Wu, Wei Qian.

**Funding acquisition:** Yun Chen, Chaoer Wu, Wei Qian.

**Investigation:** Yun Chen, Chaoer Wu, Wei Qian.

**Methodology:** Yun Chen, Chaoer Wu, Wei Qian.

**Project administration:** Yun Chen, Chaoer Wu, Wei Qian.

**Resources:** Yun Chen, Chaoer Wu, Wei Qian.

**Software:** Yun Chen, Chaoer Wu, Wei Qian.

**Supervision:** Yun Chen, Chaoer Wu, Wei Qian.

**Validation:** Yun Chen, Chaoer Wu, Wei Qian.

**Visualization:** Yun Chen, Chaoer Wu, Wei Qian.

**Writing – original draft:** Yun Chen, Chaoer Wu, Wei Qian.

**Writing – review & editing:** Yun Chen, Chaoer Wu, Wei Qian.
